# Screening and Functional Prediction of Rumen Microbiota Associated with Methane Emissions in Dairy Cows

**DOI:** 10.3390/ani14223195

**Published:** 2024-11-07

**Authors:** Jiatai Bao, Lei Wang, Shanshan Li, Jiahe Guo, Pan Ma, Xixia Huang, Gang Guo, Hailiang Zhang, Yachun Wang

**Affiliations:** 1 Laboratory of Animal Genetics, Breeding and Reproduction, Ministry of Agriculture of China, National Engineering Laboratory of Animal Breeding, State Key Laboratory of Animal Biotech Breeding, College of Animal Science and Technology, China Agricultural University, Beijing 100193, China; zhanghao9372@gmail.com (J.B.); wledu2016@163.com (L.W.); lishsh@cau.edu.cn (S.L.); heekuo@163.com (J.G.); 2 College of Animal Science, Xinjiang Agricultural University, Urumqi 830052, China; pmahem@163.com (P.M.); au-huangxixia@163.com (X.H.); 3 Beijing Sunlon Livestock Development Company Limited, Beijing 100029, China; guogang2180@126.com

**Keywords:** dairy cattle, methane emission, microorganism, rumen, Holstein

## Abstract

Agricultural activities are a significant contributor to global greenhouse gas emissions, accounting for 14.5% of total anthropogenic emissions. Specifically, greenhouse gas emissions from beef cattle and dairy cattle constitute 35% and 30% of total global livestock emissions, respectively. This study focuses on dairy cattle, exploring the complex relationships between rumen microbiota and methane emission. The methane emissions of 968 lactating Holstein cows were measured using a laser methane detector (LMD, Shanghai Hesai Technology Co., Ltd., Shanghai, China). Among the measured cows, 107 individuals were further selected into high (HME) and low methane-emitting (LME) groups, including 50 cows in the HME group and 57 in the LME group. This study analyzed differences in rumen microbiota and microbial functions between cows with varying levels of methane emissions. The results showed significant differences in the Simpson and Pielou indices of rumen bacterial communities between the HME and LME groups. Beta diversity analysis revealed significant differences in microbial community structure between the two groups. It was found that the abundance of *Bacteroidales* and *Prevotellaceae* in the rumen of cows in the HME group cows was significantly higher than that of cows in the LME group (LDA > 3, *p* < 0.05). Additionally, bacterial functions related to biosynthesis and carbohydrate metabolism were more active in the HME group. This study revealed distinct differences in the rumen bacterial communities between HME and LME cow in Chinese Holstein cattle, and identified specific bacteria and their functional differences in the HME group. The microbial characteristics and metabolic pathways provide new insights for developing strategies to reduce methane emissions, supporting the sustainable development of the dairy industry.

## Simple Summary

Agricultural greenhouse gas emissions account for 14.5% of global anthropogenic emissions, with beef and dairy cattle contributing 35% and 30% of global livestock emissions, respectively. This study focuses on dairy cattle, exploring the relationships between rumen microbiota and methane emission. Using a laser methane detector (LMD), methane emissions from 968 lactating cows were measured, and 107 cows were selected for high and low emission groups. The results showed that the abundance of *Bacteroidales* and *Prevotellaceae* in the rumen of high methane-emitting cows was significantly higher than that in the low methane-emitting cows. Additionally, it was found that bacterial functions related to biosynthesis and carbohydrate metabolism were more active in the high methane-emitting cows. These findings provide new insights for developing strategies to reduce methane emissions, supporting the sustainable development of the dairy industry.

## 1. Introduction

Agricultural greenhouse gas emissions account for 14.5% of total anthropogenic emissions globally, highlighting the urgency of addressing emissions from livestock production systems [1]. Among them, the greenhouse gas emissions from beef cattle and dairy cattle account for 35% and 30% of total global livestock emissions, respectively [2]. Given that methane has a half-life of about 10 years compared to nearly 100 years for CO_2_, reducing methane emissions is considered one of the most immediate strategies for slowing the rate of global warming [3,4].

In recent decades, genetic breeding researchers have attempted to carry out large-scale methane measurements using various methods in dairy cattle populations. Among the measurement methods, laser methane detection (LMD) is suitable for large-scale measurements due to its simplicity of operation, wide measurement range, and relatively reliable data. LMD has been used to conducted multiple studies in dairy cattle, demonstrating that the results from this device are relatively reliable [5,6]. For example, Sorg et al. [5] conducted a consistency study using four respiratory chambers and four LMD devices. A high level of consistency was observed both between different LMD devices and between the LMD method and the respiratory chamber. The correlation coefficient (R^2^) between LMD and the respiratory chambers was approximately 0.9 in four cows. The studies by Pereira [7], Kang [8], and Pickering [9] measured methane emission phenotypes of dairy cows using the LMD method. These studies estimated genetic parameters, conducted genome-wide association analyses, and performed genomic predictions for methane emission traits based on LMD phenotypes. By employing the LMD method, these studies not only provided insights into the genetic aspects of methane emissions but also demonstrated the utility of LMD-derived phenotypes in genetic evaluations.

Methane emissions from ruminants account for approximately 17% of global anthropogenic methane emissions [10]. In the rumen of ruminants, methane is produced through the anaerobic fermentation of hydrolyzed dietary carbohydrates by microorganisms [11]. Previous studies have demonstrated that methane emissions are largely influenced by the composition of the ruminal microbiota, feed intake, diet composition, physiology, and genetics [12,13,14,15,16]. Significant differences in the diversity of rumen microbial communities have been observed between dairy cows with high and low methane-emitting groups [17]. Hydrogen and carbon dioxide are the main substrates for methane production, linking microbial fermentation processes and methanogenic archaea to methane synthesis.

In Danish Holstein cattle, Difford et al. [18] conducted a study on the relationship between methane emissions, rumen microbiota, and host genetics. The results indicated that the contribution of rumen microbiota to the methane emission phenotype was 13%. The study selected the top 10% and bottom 10% of the cows based on their methane emission phenotypes and analyzed the differences in their rumen microbial communities. Although no distinct clusters were observed, the β-diversity between the two groups showed significant differences in the analysis. Similarly, Stepanchenko et al. [19] selected groups of five high and five low methane-emitting cows from a sample of 130 US Holstein cows, and used high-throughput sequencing to analyze differences in their rumen microbiota composition. It was found that the low methane-emitting cows had a relatively high abundance of *Succinivibrionaceae* and *Veillonellaceae* in their rumens. In the study of Difford et al. [18], selecting cows with high and low methane-emitting levels from multiple farms ensured selection accuracy but failed to control for differences in diet and microenvironment. In addition, Wallace et al. [20] found that differences in rumen microbial function between high and low methane-emitting beef cattle are primarily reflected in the metabolism of acetate and pyruvate. Complementing this, Martínez-Alvaro et al. [21] discovered that high methane-emitting beef cattle have a greater abundance of microorganisms associated with fiber degradation and short-chain fatty acid (such as butyrate) production, suggesting these metabolites may influence methane emissions. Additionally, studies based on small sample sizes may not fully represent the characteristics of the overall samples. It is essential to apply strict criteria when selecting samples, ensuring that all factors influencing methane emissions in dairy cows are controlled as much as possible. Currently, there are no reported studies investigating the association between rumen bacterial composition and methane emission phenotypes in Chinese Holstein cattle. This gap highlights the need for future research to explore this relationship, ideally with a well-controlled and sufficiently large sample size to provide more representative and accurate insights.

This study screened cows with different levels of methane emissions from a dairy farm sharing the same microenvironment and raised under uniform management conditions. The main objective was to analyze the differences in the composition and functionality of rumen microorganisms in lactating cows with different methane emission levels, and to identify potential microbial targets that could help reduce methane emissions in the dairy industry.

## 2. Materials and Methods

### 2.1. Animals and Methane Emission Phenotyping

The animals used in this study were from a large dairy farm in Beijing, China. The farm has approximately 3000 Holstein cattle, including around 1500 lactating cows. The cows are housed in free stalls, fed a total mixed ration (TMR), and collectively milked in a milking parlor.

The methane emission phenotypes of 968 lactating cows from this farm were measured using a hand-held laser methane detector (HS4000, Shanghai Hesai Technology Co., Ltd., Shanghai, China), with four units employed. Briefly, the device utilizes tunable diode laser absorption spectroscopy with an indium–gallium–arsenide laser at 1653 nm, targeting the absorption band of methane. The reflection of the laser beam is measured to determine the methane concentration (in ppm × m). It operates between −17 and 50 °C and 30 and 90% humidity. The accuracy of pointing the device at a methane source is facilitated by a second, visible green helium-neon pointing laser (532 nm) next to the invisible measuring laser. The HS4000 connects via Bluetooth to a smartphone or tablet running the HESAI app (v1.0.5, Shanghai Hesai Technology Co., Ltd.) for data export and storage. Each cow is measured once in the morning and once in the afternoon, for two consecutive days in each barn. Each measurement session lasts three minutes, generating approximately 180 data points per measurement.

The raw data file is first processed to remove outliers below 200 or above 8000 ppm × m (determined based on preliminary experiments and observed data patterns). A threshold is then set at the mean plus a standard deviation, and only data points above this threshold are retained to calculate the average as the methane emission phenotype for corresponding measurement. As a further quality control, cows with at least two measurements are retained, and the mean of these measurements is taken as the final methane emission phenotype for each cow during the experiment. The final methane emission phenotype of each cow is performed quality control using a mean ± 3 standard deviations method. Finally, a total of 648 cows with usable methane emission phenotypes were available, and production information for these 648 cows was collected from farm management software (v6.x).

### 2.2. Methane Emission Grouping and Sample Collection

Among 648 phenotyping cows, animals producing 30–40 kg of milk per lactation with one to two parities were selected. According to a criterion of mean ± 0.65 standard deviations of methane emission phenotypes, 57 low methane-emitting (LME) and 50 high methane-emitting (HME) cows were selected into an LME group and HME group, respectively. Rumen fluid samples were collected within one week of completing the phenotypic measurements, and the collection was carried out over two mornings. To minimize the risk of cross-contamination and saliva contamination, we implemented a strict cleaning protocol. After each sample collection, the cannula was rinsed thoroughly with water using a syringe, followed by two air flushes. A new syringe was used for each cow, and the first 20 mL of rumen fluid was discarded to ensure sample integrity. The remaining fluid was filtered and placed into sterile 5 mL cryotubes, then quickly frozen in liquid nitrogen until DNA extraction.

### 2.3. DNA Extraction, Library Construction, and Sequencing

DNA was extracted from rumen fluid samples using the E.Z.N.A.^®^ Soil DNA Kit (Omega Bio-tek, Norcross, GA, USA) and assessed for concentration, purity, and quality using a spectrophotometer (NanoDrop 2000, Thermo Fisher Scientific Inc., Waltham, MA, USA) and 1% agarose gel electrophoresis. The specific primers 338F and 806R were used to amplify the V3-V4 regions of bacterial 16S rRNA via PCR, which were then evaluated using 2% agarose gel electrophoresis. The PCR products were purified using the AxyPrep DNA Gel Extraction Kit (Corning Inc., Union City, CA, USA) and sequenced on the Illumina MiSeq platform (Illumina Inc., San Diego, CA, USA) using PE2*300 paired-end sequencing. DNA extraction, PCR amplification, fluorescence quantification, and MiSeq library construction were performed at Shanghai Major Bio-pharm Technology Co., Ltd. (Shanghai, China).

### 2.4. Sequencing Data Processing and Analysis

Bacterial 16S rRNA sequences were processed using Qiime2 (v2023.9) [22], including integration, quality control, and denoising, and finally clustering into Amplicon Sequence Variants (ASVs). In the Qiime2 environment, consensus sequences were generated for each ASV, followed by data exportation and further quality control. ASVs with a total abundance of less than five ASVs were filtered out, and their corresponding representative sequences were excised. Taxonomic annotation of ASVs was performed using the Greengenes database (gg_13_8_otus) with a confidence threshold of 0.8.

To explore the functional pathways of rumen microorganisms between LME and HME groups, KEGG orthologs were predicted for each ASV using PICRUSt2 (2.3.0) [23] software. Secondary and tertiary functional classifications based on the KEGG database were then statistically analyzed. For the subsequent visualization analyses, the packages MicrobiotaProcess (v1.14.0) [24] and microeco (v1.7.1) [25] in R (v4.3.1) were mainly used.

In this study, the *t*-test function in R was used to analyze methane emissions, milk production, and bacterial α diversity indices (Simpson [26], Chao1 [27], Observe, Pielou [28]) between the two groups. To ensure that the sequencing depth was sufficient to capture the diversity of the rumen microbial communities, we performed an alpha rarefaction curve analysis. The results showed that microbial diversity reached a plateau as the sequencing depth increased (Figure S1). Based on the amplicon data, Principal Coordinates Analysis (PCoA) of rumen bacteria from both groups was tested using the ADONIS method, and Spearman correlations were calculated for the top 10 phyla and genera between the two groups. The LEfSe method [29] (Linear Discriminant Analysis Effect Size) was used to compare significant differences in bacterial taxa of rumen between two groups and *p*-values were adjusted using the False Discovery Rate (FDR) method (log10(LDA) > 3 & FDR < 0.05). Bacterial KEGG Orthology (KO) terms and pathways predicted by PICRUSt2 were normalized to Reads Per Kilobase Million (RPKM). Differential functional pathways were screened using the LEfSe method (log10(LDA) > 1 & FDR < 0.05).

## 3. Results

### 3.1. Phenotypic Characteristics of Methane Emissions in Dairy Cows

In this study, methane emission data for 648 dairy cows were obtained after data quality control. The average methane emission concentration for the 648 cows was 853.25 ppm × m, with a standard deviation of 284.01 ppm × m, and a variation coefficient of 33.29%, indicating substantial inter-individual variability. The average methane emission concentration for LME cows was 628.80 ppm × m, with a standard deviation of 78.61 ppm × m, and a coefficient of variation of 12.50%. For HME cows, the average concentration was 1134.21 ppm × m, with a standard deviation of 209.58 ppm × m, and a variation coefficient of 18.51% (Table 1).

**Figure 1:**
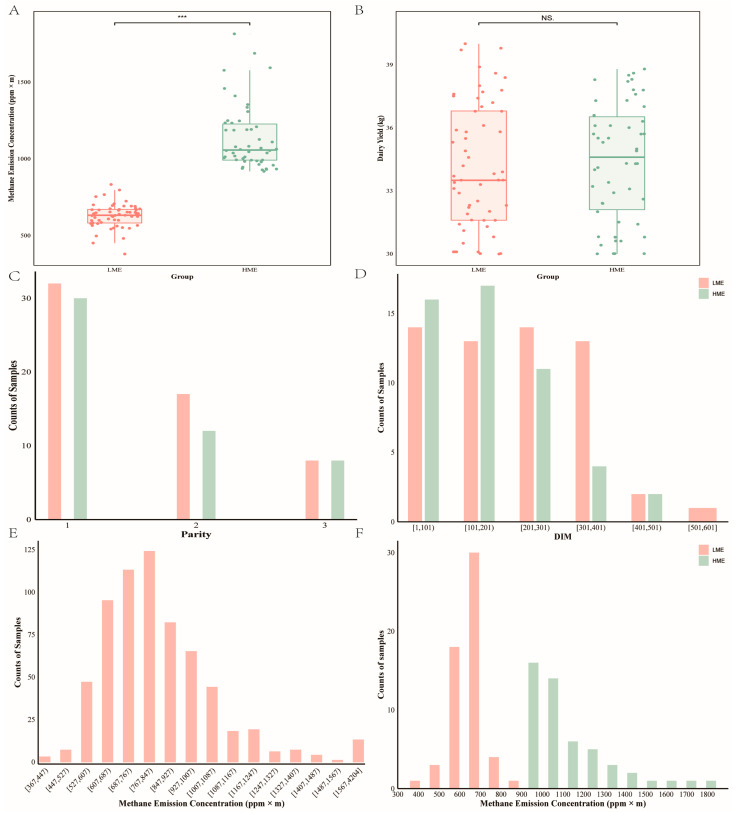
Overview of phenotype characteristics for the selected low methane-emitting (LME) and high methane-emitting (HME) groups. (**A**) Differences of methane emission concentrations for the two groups and their *t*-test analysis results; (**B**) Differences of milk yield for the two groups and their T-test analysis results; (**C**) Distribution of parities for the two groups; (**D**) Distribution of days in milk (DIM) for the two groups; (**E**) Distribution of methane emission for all cows after quality control; (**F**) Distribution of methane emission for the two groups. Symbols indicate significance (***, *p* < 0.001; NS, *p* > 0.05).

Among 648 phenotyping cows, 107 were selected based on their methane emission concentrations, with 57 in the LME group and 50 in the HME group. As shown in Figure 1, the methane emission concentrations of the 648 cows followed an approximately normal distribution, and a highly significant difference in methane emission concentrations was found between the LME and HME groups (*p* < 0.001), with no significant difference in milk yield. Additionally, the distribution of parity and days in milk was relatively even between the cows in both groups.

### 3.2. Ruminal Microbial Composition and Differential Analysis Between LME and HME Groups

**Figure 2:**
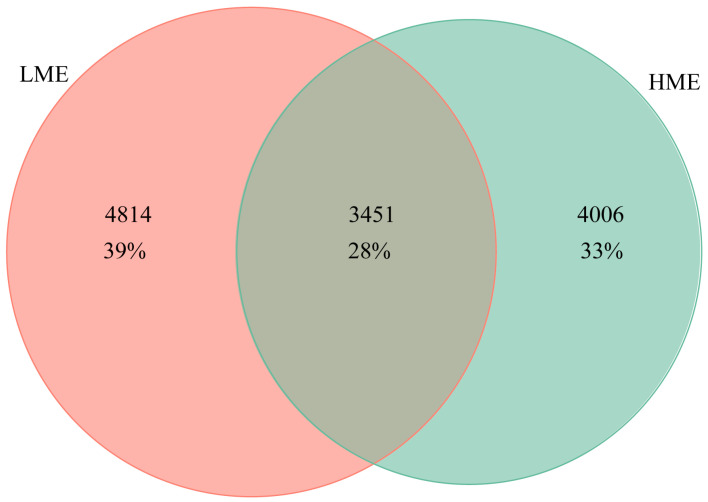
The number of ASVs (Amplicon Sequence Variants) detected in the low methane-emitting (LME) and high methane-emitting (HME) groups of cows.

In total, 5,727,096 raw sequence reads were obtained for the rumen fluid samples of 107 dairy cows. After assembly, quality control and chimera removal, 1,817,692 high quality sequences were retained, with an average of 16,987.78 sequences per sample. In this study, the shared and unique microbial communities between the two groups are shown in Figure 2. The LME group harbored 4814 unique ASVs, while the HME group contained 4243 unique ASVs. In addition, 3451 ASVs were detected in both groups, representing 28% of the total ASVs. The number of ASVs in the rumen of cows from the LME group is higher than that in the cows of HME group.

### 3.3. Microbial Diversity Analysis of LME and HME Groups

**Figure 3:**
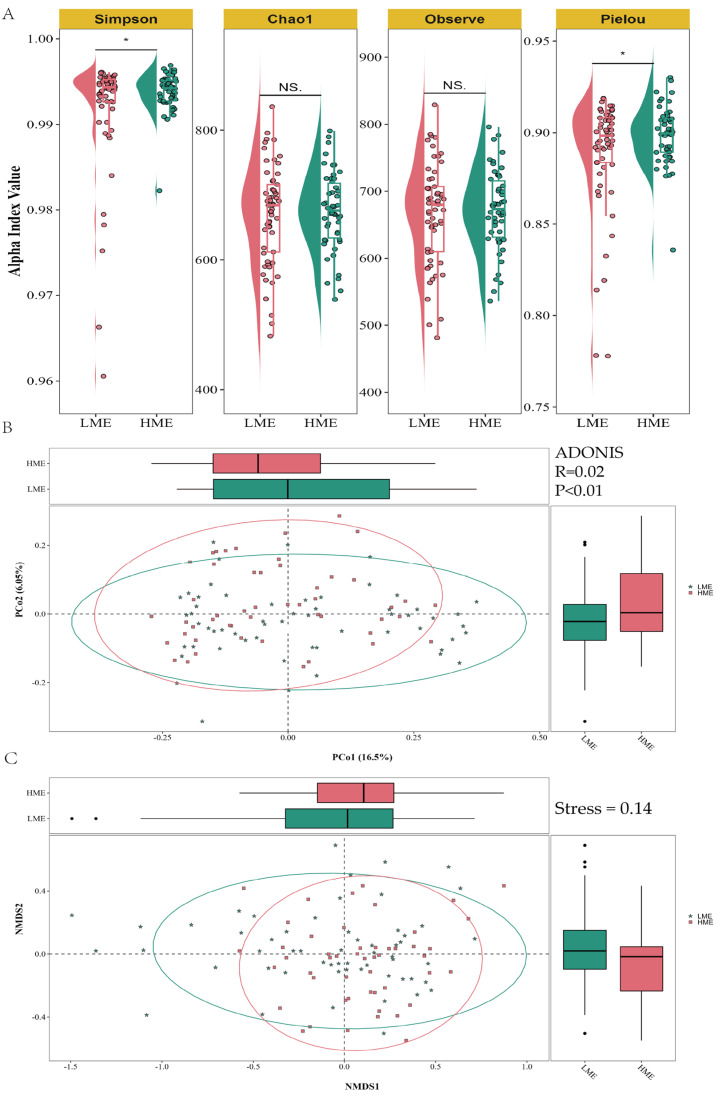
Microbial diversity analysis of rumen in the low methane-emitting (LME) cows and high methane-emitting (HME) cows. (**A**) Shannon index, Chao1 index, Observe index, and Pielou index based on the abundance of ASVs; (**B**) Principal Coordinates Analysis (PCoA); (**C**) Non-metric Multidimensional Scaling (NMDS). * represents *p* <  0.05, and NS represents *p* > 0.05.

This study conducted a comparative analysis of the alpha diversity indices of ruminal microbiota in LME group and HME groups of cows (Figure 3A). We utilized the Simpson, Chao1, Observed, and Pielou indices to measure species diversity and evenness, and performed *t*-tests on the diversity indices between the two groups of cows. The comparison of the Simpson index indicated that the LME group had a higher species diversity compared to the HME group (*p* < 0.05), whereas no significant differences were observed for the Chao1 and Observed indices. The Pielou index suggested that the LME group had lower species evenness than the HME group (*p* < 0.05).

To investigate the differences of microbial community structure in rumen between the LME and HME groups, Principal Coordinates Analysis (PCoA) and Non-metric Multidimensional Scaling (NMDS) were employed in this study. As shown in Figure 3B, PCoA revealed subtle differences in the microbial community structure between two groups, with the first principal coordinate (PCo1) accounting for 16.5% of the community variation. Although the R-squared value was only 0.02, indicating limited explanatory power for the community structure differences, these differences were statistically significant as determined by the ADONIS test (R = 0.02, *p* = 0.01).

We further utilized NMDS analysis to dimensionally reduce the distribution characteristics of the ruminal microbiota community structure in the two groups. The results showed a stress value of 0.14, indicating that the analysis was reliable and accurately reflected the structure of the original data (Figure 3C). Despite substantial overlap in the NMDS space, a certain degree of separation between the samples from the two groups was observed. Accompanied by elliptical confidence intervals, it was observed that despite some overlap, the central tendencies of the two groups were distinct, suggesting significant differences of microbial community structure in the cow rumen with different methane emission levels.

### 3.4. Dominant Species Composition in the Rumen Between Low and High Methane-Emitting Cows

**Figure 4:**
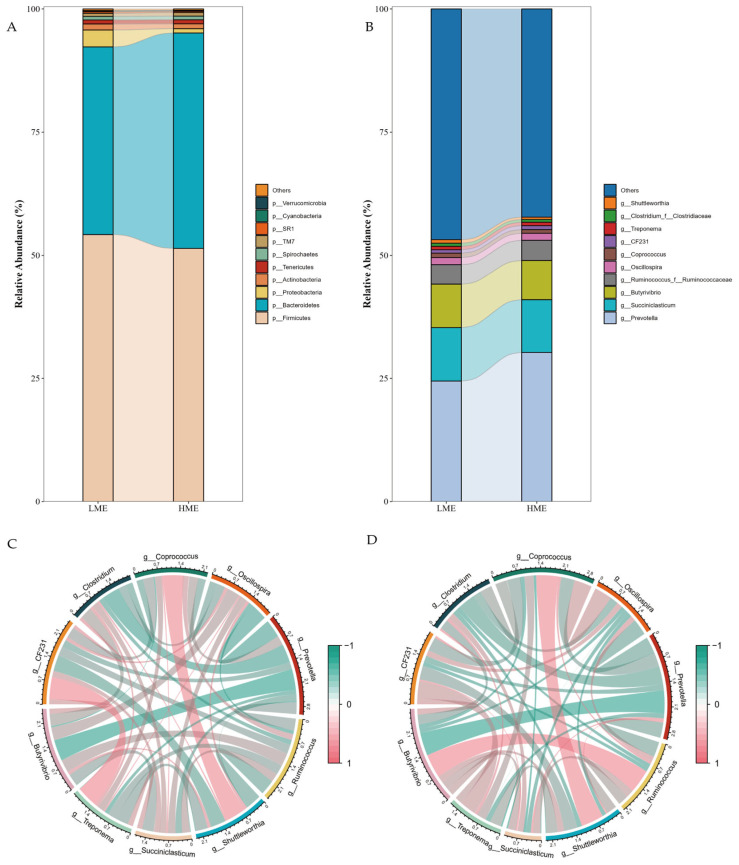
The composition of dominant species in the rumen of low methane-emitting (LME) and high methane-emitting (HME) cows, along with a correlation analysis of dominant genera between the two groups. (**A**) Horizontal species composition accumulation map of the top 10 phyla; (**B**) Horizontal species composition accumulation map at the genus level; (**C**) Spearman correlation of dominant genera in the rumen of LME cows; (**D**) Spearman correlation of dominant genera in the rumen of HME cows.

At the phylum level, 29 phyla were identified, among which 7 dominant phyla had an average relative abundance greater than 1%, namely *Firmicutes*, *Bacteroidetes*, *Proteobacteria*, *Actinobacteria*, *Tenericutes*, *Spirochaetes*, and *TM7*. These seven dominant phyla accounted for more than 95% of the total bacterial abundance. *Firmicutes* and *Bacteroidetes* dominated the rumen of cows in both groups, with relative abundances of 54.24% and 38.06% in the LME group, and 51.43% and 43.68% in the HME group, respectively (Figure 4A). Differences in the relative abundance of *Proteobacteria*, *Actinobacteria*, *Tenericutes*, *Spirochaetes*, *TM7*, *SR1*, *Cyanobacteria*, and *Verrucomicrobia* were observed between the two groups.

At the genus level, 86 bacterial genera were identified, among which 12 genera had an average relative abundance greater than 1%. *Prevotella*, *Succiniclasticum*, and *Butyrivibrio* were the most dominant genera, with relative abundances of 24.46% and 30.26%, 10.85% and 10.70%, and 8.82% and 8.02% in the LME and HME groups, respectively (Figure 4B). Genera from the family *Ruminococcaceae*, such as *Ruminococcus*, *Oscillospira*, and *Coprococcus*, although present at lower relative abundances, also showed differences between the two groups.

To explore the interactions among the dominant genera, we conducted a correlation analysis of the relative abundances of the top ten genera in the rumen of cows in the LME and HME groups, and constructed circular correlation charts. In the LME group, several genus pairs showed significant positive correlations, notably a strong positive correlation between *Prevotella* and *Succiniclasticum* (Figure 4C). In the HME group, a positive correlation was observed between *Butyrivibrio* and *Ruminococcus*, but a strong negative correlation was found with *Prevotella* (Figure 4D).

### 3.5. Analysis of Differences in the Rumen Microbiota of Cows Between the LME and HME Groups

**Figure 5:**
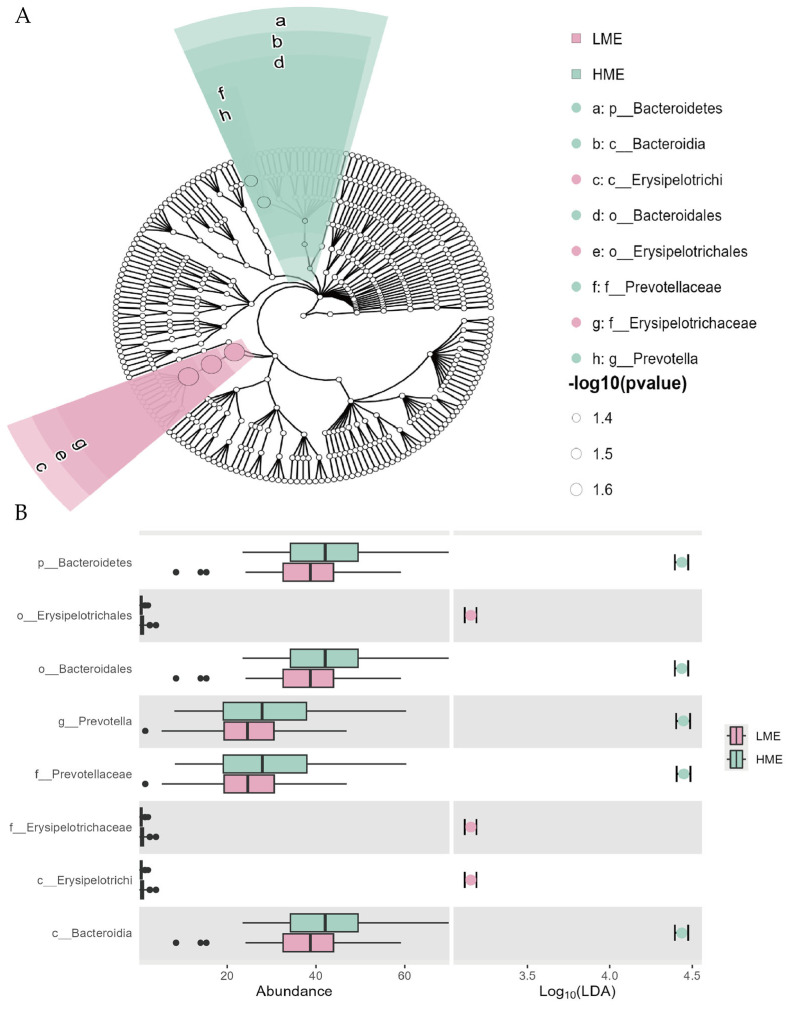
Different structures of rumen microbiota of cows in the low methane-emitting (LME) and high methane-emitting (HME) groups. (**A**) Cladogram of analysis demonstrated microbiome differences of the two groups at various phylogenic levels. Circles from the inside out indicate the phylogenetic levels from the phylum to genus; (**B**) The abundance of the differential taxa and the magnitude of the LDA effect. FDR < 0.05 & log10(LDA) > 3.0.

We used the LEfSe method to analyze the significantly different microbial taxa in the rumen between LME and HME cows, cladograms and histograms of taxa with are shown in Figure 5A,B. In total, one, two, two, two, and one differentially abundant taxa were identified at the phylum, class, order, family, and genus levels, respectively (log10|LDA| > 3 & FDR < 0.05). The results showed that at the phylum level, the abundance of *Bacteroidetes* in the rumen was significantly different between the LME and HME cows. At the order level, the abundance of *Erysipelotrichales* and *Bacteroidales* also showed significant differences between two groups. Additionally, at the family level, the abundances of *Prevotellaceae* and *Erysipelotrichaceae* showed significant intergroup differences, with *Erysipelotrichaceae* being more abundant in the LME cows than that in the HME cows. At the genus level, the abundance of *Prevotella* was significantly higher in the HME group than in the LME group.

### 3.6. Functional Profile Analysis of the Rumen Flora

**Figure 6:**
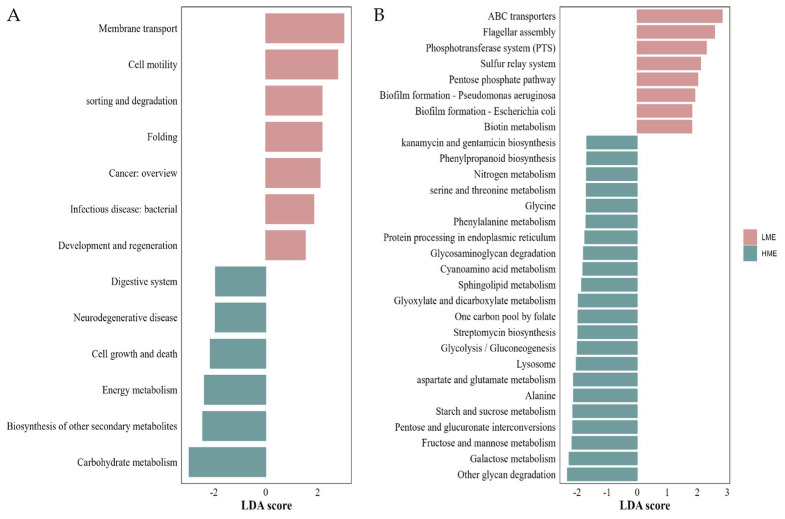
Significantly different KEGG functional pathways in the rumen bacteria between the low methane-emitting (LME) and high methane-emitting (HME) cows. (**A**) Differential KEGG Level-2 pathways in the rumen bacteria between the LME and HME groups; (**B**) Differential KEGG Level-3 pathways in the rumen bacteria between the LME and HME groups. FDR< 0.05 & log10|LDA| > 1.0.

We utilized the PICRUST2 software (v2.3.0) to predict the KEGG functional pathways of rumen bacteria. A total of 47 level-2 pathways and 347 level-3 pathways were identified. The significant differences in KEGG level-2 pathways are shown in Figure 6A. Seven functional pathways were enriched in the LME group, while six functional pathways were enriched in the HME group. membrane transport, cell motility, sorting and degradation, folding, and cancer: overview were significantly enriched in the LME group (log10|LDA| > 2 & FDR < 0.05). The HME group showed significant enhancement in neurodegenerative diseases, cell growth and death, energy metabolism, biosynthesis of other secondary metabolites, and carbohydrate metabolism (log10|LDA| > 2.0 & FDR < 0.05). The differential level-2 pathways were mainly related to metabolic systems, immune systems, digestive systems, and energy systems.

The LME group had eight enriched level-3 functional pathways, whereas the HME group had 22 enriched level-3 functional pathways (Figure 6B). The significantly enriched metabolic pathways in the HME group included glyoxylate and dicarboxylate metabolism; one carbon pool by folate; streptomycin biosynthesis; glycolysis/gluconeogenesis; lysosome; aspartate; and glutamate metabolism; alanine, aspartate, and glutamate metabolism; starch and sucrose metabolism; pentose and glucuronate interconversions; fructose and mannose metabolism; galactose metabolism; and other glycan degradation (log10|LDA| > 2 & FDR < 0.05). The LME group showed significant increases in cell membrane transport systems and cell motility (log10|LDA| > 2 & FDR < 0.05), such as ABC transporters, flagellar assembly, and the phosphotransferase system (PTS). The differences in level-2 and level-3 functions between the LME and HME groups indicate that cows in the HME group are more active in substance synthesis and carbohydrate metabolism, while cows in the LME group are more active in cell membrane transport systems and cell motility.

## 4. Discussion

Due to the methodological constraints in quantifying methane emissions from ruminants, measuring methane emissions in large populations remains uncommon [30], leading to studies of emission traits in dairy cattle being predominantly restricted to small cohorts. This study utilized advanced portable devices to perform extensive measurements of methane emission concentrations across a significant sample of dairy cattle. To minimize measurement errors, we ensured a sufficient number of replicates and maintained a measurement duration of over 3 min. We utilized the same LMD equipment and confirmed the device’s accuracy by using a 1% CH4 standard gas before each measurement. The composition and diversity for the rumen microbial community of Holstein cows at different emission levels were systematically assessed. Additionally, leveraging data from a substantial sample size, this study further explored the correlations between the rumen microbiota and their methane emission levels in Holstein population.

This study was conducted to examine the consistency between laser methane detector (LMD) readings and respiratory chamber measurement data, finding good alignment between the two methods, which holds biological significance [31]. Ricci et al. studied the correlation between measurements obtained using LMD and respiration chambers in 24 lactating ewes and 72 finishing bulls, finding that the R^2^ value for the correlation between LMD and respiration chambers reached as high as 0.86 [32]. Considering the necessity for evaluating large population, we selected the LMD as our measuring tool and conducted comprehensive methane emission concentration measurements across approximately 1000 lactating cows from a dairy farm. Through rigorous data selection and quality control, we obtained methane emission concentration data for 704 Holstein cows. The mean methane emission concentration was 840.61 ppm × m with a standard deviation of 203.33 ppm × m, which was different from the methane emission concentrations reported in most dairy cattle studies [7,32,33], a discrepancy that may be attributed to the precision of our measuring equipment. Since our measurements infrequently captured methane concentrations during respiration, our study considered the methane concentrations emitted through belching as the definitive metric for the methane emissions of dairy cattle.

We performed an Amplicon Sequence Variant (ASV) clustering analysis to explore microbial differences between LME and HME cows. The analysis showed that only 28% of ASVs were shared, indicating notable variations in microbial community composition between the two groups. The alpha diversity metrics provided a more precise validation, encapsulating the structural complexity of the ecological communities, including richness (the number of taxa) and evenness (the distribution of abundance across taxa) [34]. In this study, the Simpson and Pielou indices indicated that the gut microbiota diversity of the LME group was higher than that of the HME group, but with lower evenness. A previous study by Granja-Salcedo et al. reported similar variations in microbial community diversity between cattle with different methane emission levels [35]. There are various beta diversity measures that can quantify the differences between different sample compositions for clustering, and using different beta diversity measures produces different clusters and makes it difficult to choose between them [36]. Thus, we utilized unweighted UniFrac analysis to compare the similarities and heterogeneities of the gut microbial communities between the LME and HME groups. While no distinct clustering pattern was observed, the analysis revealed considerable differences in the rumen microbial composition between the two groups. This significant disparity in rumen microbiota structure across varying methane emission levels might be related to the methane generation process and warrants further exploration. These findings align with published studies highlighting structural differences at both the bacterial and archaeal levels [37].

In our study, the dominant phyla across both groups were *Firmicutes*, *Bacteroidetes*, and *Proteobacteria*. While methanogens are recognized as the sole methane producers in the rumen, the relationship between methane production and methanogen abundance remains unclear. Some studies have shown a direct correlation between methanogen abundance and high methane emissions in ruminants, while others have found no such correlation [20,38,39]. The composition of the bacteria and eukaryotic communities is suggested to exert a large influence on the methanogenesis potential of the rumen microbiome, as methanogens depend on the activity of other microorganisms [12]. Through differential analysis, we observed that the levels of *Bacteroidetes* were significantly higher in the HME group compared to the LME group. It is well recognized that the substrates provided by ruminal microbial fermentation for methane synthesis include hydrogen, carbon dioxide, formate, acetate, and other methyl compounds [40,41], with hydrogen and CO_2_ serving as the primary substrates for ruminal CH_4_ production [42,43]. Most of the hydrogen produced during microbial carbohydrate fermentation is utilized for CH4 production [44]. Studies have shown that the majority of bacteria within the Bacteroidetes phylum are involved in polysaccharide degradation [45,46,47], resulting in the production of various organic acids as well as hydrogen and carbon dioxide. Therefore, we hypothesize that the elevated levels of Bacteroidetes in the HME group may provide more substrates for methane synthesis. Differences in certain Bacteroidetes species appear to be associated with higher methane emissions, and there are studies consistent with our findings [12]. Additionally, significant inter-group differences were observed in *Erysipelotrichales* and *Prevotellaceae*, which are consistent with those reported in sheep by Kamke et al. [48]. There have been studies reported, several non-methanogenic microbial groups have been consistently associated with high methane emission, among which are taxa from the genera *Ruminococcus*, *Prevotella* and *Mogibacterium*, as well as *protozoa* [49]. Our research reinforces the association between methane emission levels and specific microbial communities, and offers new insights into the complex interactions between rumen microbial diversity and host methane emissions.

To investigate the impact of varying methane emission levels on rumen microbial functions, we utilized PICRUSt2 to predict their potential functions. Annotations of the inferred gene families were made based on the KEGG database, integrating pathways from level 2 to level 3. Our findings revealed functional changes in the rumen microbiota across different methane emission levels. LDA differential analysis indicated that cattle from the HME group exhibited more active synthesis of substances and carbohydrate metabolism, whereas the LME group showed greater activity in cellular membrane transport systems and cell motility. In the HME group, the enhanced involvement of rumen microbiota in energy metabolism processes led to increased methane production. This process requires the breakdown of substantial substrates to provide key precursors for methane production, such as hydrogen and carbon dioxide. In cows with high methane emissions, the bacterial functions in aspartate and glutamate metabolism, alanine metabolism, and other glycan degradation are more pronounced. The metabolism of aspartate and glutamate produces carbon dioxide and hydrogen gas, while rumen microbes metabolize these amino acids into short-chain fatty acids and gaseous products [50]. The metabolism of alanine generates pyruvate, which can further be metabolized into carbon dioxide, hydrogen gas, and volatile fatty acids [51]. The degradation of cellulose and hemicellulose represents the primary fermentation pathways of rumen microbes, resulting in significant production of acetate and butyrate, along with substantial release of carbon dioxide and hydrogen gas [52]. Consequently, the potential mechanism for higher methane emissions may involve these bacteria supplying more substrates for methanogens through the aforementioned metabolic pathways, leading to increased methane production. These microbes also more effectively utilize their ecological niches, including the use of diverse organic materials as energy sources, thereby supporting extensive metabolic activities [53]. Moreover, the physical and chemical conditions of the rumen environment, along with host management and genetic factors, also influence the functionality of the microbial community [54,55]. Therefore, the characteristics of the microbial community in the HME group likely reflect their adaptation to physiological and environmental conditions, demonstrating efficient strategies in energy production and nutrient transformation, ultimately leading to higher methane emissions.

Our findings provide valuable insights into the interactions between methane emissions in dairy cattle and the underlying microbial and metabolic processes, suggesting potential interventions for mitigating greenhouse gas emissions. Based on our research, we propose the development of microbial preparations or more efficient cellulase and hemicellulase enzymes to enhance the complete fermentation of cellulose in the rumen, thereby potentially reducing methane production.

However, it is important to note that the 16S rRNA gene sequencing employed in this study did not include archaea, fungi, protozoa, or their interactions, which limits our understanding of the complete rumen microbiome. Additionally, there is room for improvement in our phenotypic measurement methodologies.

To address these gaps, we plan to conduct further experimental validation by collecting rumen fluid samples and establishing an in vitro rumen fermentation model. This approach aims to confirm the accuracy of the characteristic microorganisms identified in our study. Furthermore, we intend to explore the relationship between these characteristic microbes and associated metabolites with host genetic factors to deepen our understanding of these complex interactions. These topics will be the focus of our future research efforts.

## 5. Conclusions

This study revealed the taxonomic diversity and functional differences of rumen microbial communities between low and high methane-emitting cows, and the significant differences in the composition and function of rumen microbiota were observed. These microbial communities may be closely related to the methane emission characteristics of dairy cows. *Prevotella* was significantly enriched in the rumen of high methane-emitting cows. Bacterial functions related to biosynthesis and carbohydrate metabolism were significantly enriched in the high methane-emitting cows, which may indicate that the rumen of high methane-emitting cows has more active feed fermentation and carbohydrate metabolism, with more active energy metabolism. This study contributes to the understanding of rumen microbial differences between low and high methane-emitting cows and provides valuable information to elucidate the relationship between host rumen microbiota and methane emission.

## Figures and Tables

**Table 1:** Descriptive statistics of methane emissions for cows in the low methane-emitting (LME) cows, high methane-emitting (HME) cows, and all cows after quality control.

Group	Mean (ppm × m)	SD (ppm × m)	Max (ppm × m)	Min (ppm × m)	CV
LME (57)	628.80	78.61	832.75	379.42	12.50%
HME (50)	1134.21	209.58	1814.73	918.13	18.51%
ALL (648)	853.25	284.01	4203.75	367.18	33.29%

## Data Availability

The data that support the findings of this study are available from the corresponding author, Yachun Wang, upon reasonable request.

## Supplementary Materials

The following supporting information can be downloaded at: https://www.mdpi.com/article/10.3390/ani14223195/s1, Figure S1: Alpha diversity rarefaction curve in the low methane-emitting (LME) and high methane-emitting (HME) groups.
