# Supplementary figures and images for: Screening and Functional Prediction of Rumen Microbiota Associated with Methane Emissions in Dairy Cows
Source: Animals (Basel). 2024 Nov 7;14(22):3195. doi: 10.3390/ani14223195 (PMC11591143; doi:10.3390/ani14223195)

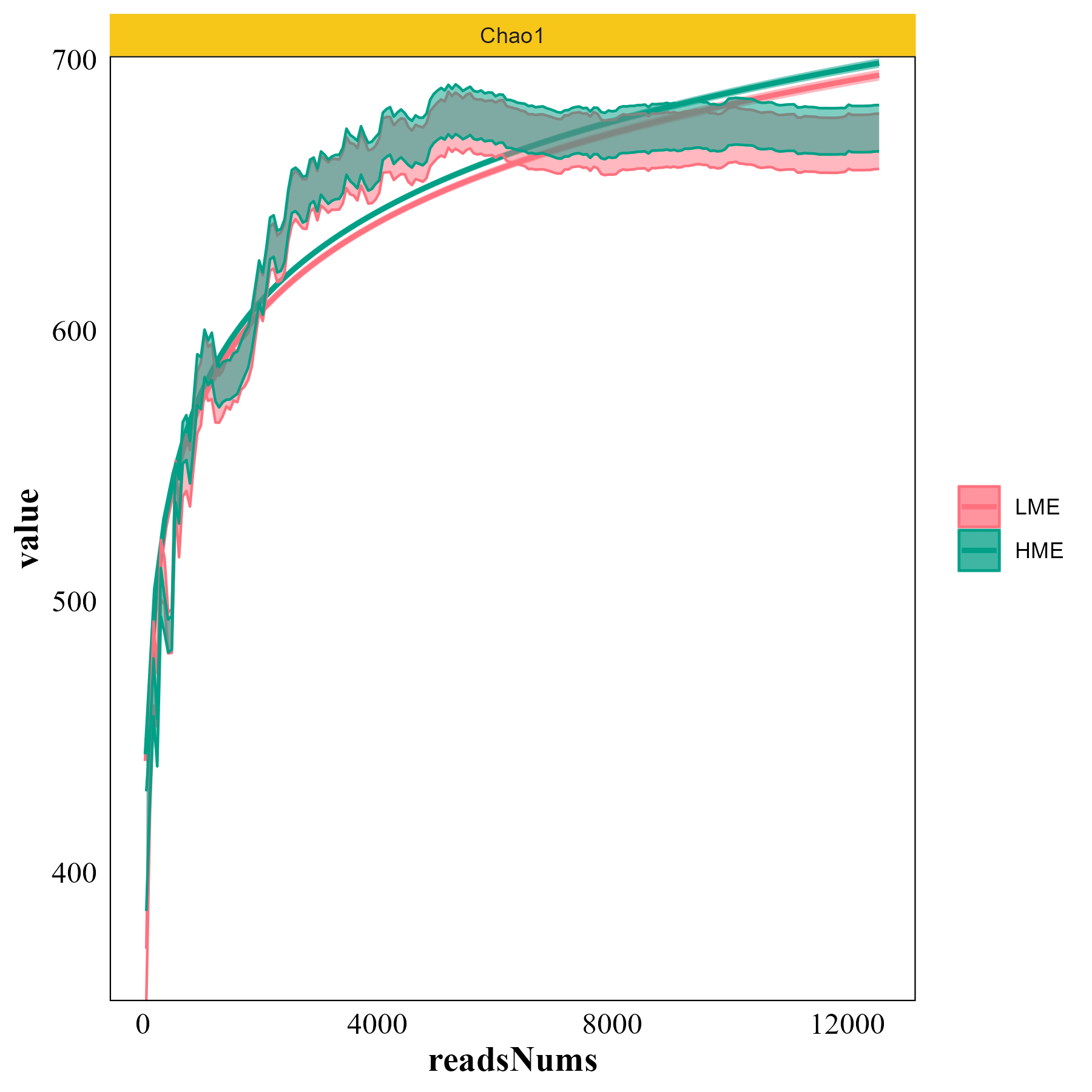

Supplement: Supplementary file 1 [file animals-14-03195-s001.zip › animals-3268966-supplementary.png]
